# Exact Bayesian inference for the detection of graft-mobile transcripts from sequencing data

**DOI:** 10.1098/rsif.2022.0644

**Published:** 2022-12-14

**Authors:** Melissa Tomkins, Franziska Hoerbst, Saurabh Gupta, Federico Apelt, Julia Kehr, Friedrich Kragler, Richard J. Morris

**Affiliations:** ^1^ Computational and Systems Biology, John Innes Centre, Norwich Research Park, Norwich NR47UH, UK; ^2^ Max Planck Institute of Molecular Plant Physiology, Max Planck Institute, Am Mühlenberg 1, Potsdam-Golm 14476, Germany; ^3^ Institute of Plant Science and Microbiology, Universität Hamburg, Ohnhorststrasse 18, Hamburg 22609, Germany

**Keywords:** Bayesian inference, mobile mRNA, long-distance transport, RNA-Seq analysis, grafting, sequencing errors

## Abstract

The long-distance transport of messenger RNAs (mRNAs) has been shown to be important for several developmental processes in plants. A popular method for identifying travelling mRNAs is to perform RNA-Seq on grafted plants. This approach depends on the ability to correctly assign sequenced mRNAs to the genetic background from which they originated. The assignment is often based on the identification of single-nucleotide polymorphisms (SNPs) between otherwise identical sequences. A major challenge is therefore to distinguish SNPs from sequencing errors. Here, we show how Bayes factors can be computed analytically using RNA-Seq data over all the SNPs in an mRNA. We used simulations to evaluate the performance of the proposed framework and demonstrate how Bayes factors accurately identify graft-mobile transcripts. The comparison with other detection methods using simulated data shows how not taking the variability in read depth, error rates and multiple SNPs per transcript into account can lead to incorrect classification. Our results suggest experimental design criteria for successful graft-mobile mRNA detection and show the pitfalls of filtering for sequencing errors or focusing on single SNPs within an mRNA.

## 1. Introduction

Hundreds to potentially thousands of different RNA molecules are transported over long distances within plants [1–3] and between parasitic plants and their hosts [4–6]. Several such transported RNAs have been shown to act ‘non-cell autonomously’ [7], playing roles in signalling and development in cells distinct from where they originated [8,9]. Determining which RNAs are transported within plants, from where to where and under which conditions, is important for evaluating their potential involvement in signalling processes [10].

Sequencing all messenger RNAs (mRNAs), using RNA-Seq, extracted from plant tissue from grafts between different species, accessions, ecotypes or cultivars, i.e. between plants with different genetic backgrounds, is a popular approach for identifying transcripts that are transported over graft junctions and potentially also over long distances within a plant [11–18]. The identification of transported transcripts is, however, not without challenges, and the overlap between existing graft-mobile mRNA classifications is poor [3]. Such inconsistencies have been suggested to be due to experimental conditions and technical differences that hinder their direct comparability [3].

**Figure 1.**
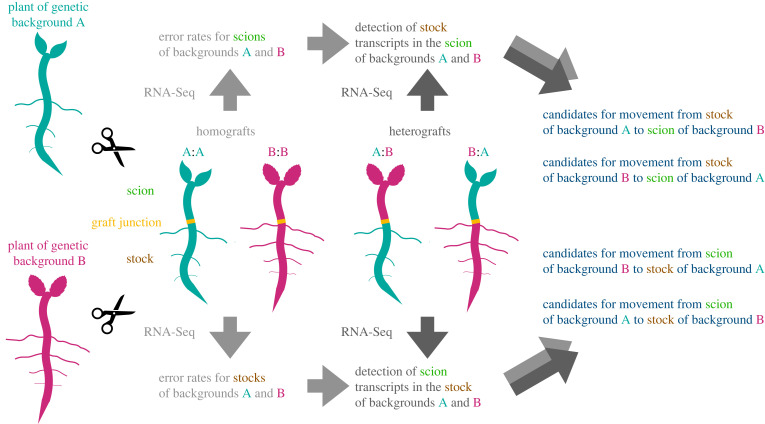
Workflow of the inference steps for determining the evidence that a transcript has travelled across a graft junction. Two plants with different genetic backgrounds are grafted in different combinations. The grafted parts are known as stock and scion. Homograft data can be used to infer the error rates per SNP in stock and scion for each genetic background. The heterograft data can then be evaluated in light of the homograft data. If the evidence for a transcript having traversed the graft junction is greater than the evidence for the data being explainable by sequencing errors, then the transcript is a candidate for being long-distance mobile.

The vegetative material that is grafted onto another plant is referred to as the scion, the plant onto which the scion is grafted is referred to as the stock, see figure 1. If a transcript that originates from the stock is found in scion tissue or a transcript from the scion is found in stock tissue, then a plausible interpretation is that it has moved over the graft junction. This inference depends on the ability to distinguish between mRNA molecules from the two grafted plants.

Homologous genes from closely related organisms often have highly similar sequences. Sequences that are identical cannot be distinguished. We assume that distinguishable homologous mRNAs from the two grafted plants with different genetic backgrounds (genotypes) differ in a number of nucleotide positions, single-nucleotides polymorphisms (SNPs). Other differences, such as insertions or deletions, are possible but not considered here.

Given that the numbers of transported transcripts are often low compared with the number of endogenous copies [18] and that sequencing errors can be expected [19,20], it is important to identify and account for potential errors in the data. In addition to sequencing errors, processing errors can arise from the differing qualities of the reference genomes, sequence divergence between the accessions used for grafting and the closest references genomes, and the level of sequence similarity between the genomes of the grafted plants.

Our goal is thus to develop a framework for assessing from which genotype transcripts have originated. If we sample tissue from one of the grafted genotypes (local), we may find SNPs associated with the other genotype (distal). A challenge is to assess whether the RNA-Seq reads for these positions that could be indicative of transcripts that have crossed the graft junction can be explained by expected biological and technical variation in the local genotype.

Here, we construct a probabilistic description of how to distinguish sequencing errors from graft-mobile transcripts using a Bayesian formalism for which we derive exact (analytical rather than numerical) solutions. Bayesian approaches [21,22] have been employed extensively in many fields, including sequence analysis and systems biology [23–31], but cases that are analytically tractable remain limited [32–34]. Analytical solutions do not require computationally expensive numerical approximations, such as Monte Carlo integration, that have become common practice in Bayesian inference. The presented approach takes read depths, SNP-specific error rates, replicates and multiple SNPs per transcript into account. We assume that the SNP positions themselves have been identified from the comparison of high-quality genomes or high-quality transcriptome data. We consider sequencing errors at those positions but do not account for uncertainty in those positions. We develop a Bayesian framework for evaluating the evidence for a transcript being graft-mobile, over the evidence for that data being consistent with sequencing or processing errors. This has the advantage that we can rank the transcripts by the data supporting their movement. We evaluate our approach using simulated data. This allows us to have confidence in the assignment as well as to examine the performance over a range of possible data properties (error rates, read depths and number of SNPs). We compare our methodology with other approaches, and based on true and false positive rates (TPR and FPR), we find that filtering SNPs with measurable errors and not taking error rates or read depths adequately into account have significant detrimental consequences on the classification performance. The proposed Bayesian approach overcomes these issues and with controlled simulated data can accurately identify graft-mobile transcripts, substantially outperforming other methods for classifying transcripts based on RNA-Seq reads. Furthermore, the Bayesian formalism can elegantly incorporate all SNPs found on each transcript. The analysis using simulated data shows that combining the information from multiple SNP positions increases classification accuracy. In fact, we find that over a large range of parameters the proposed method classifies simulated data near perfectly with an accuracy of close to 100%.

## 2. Results

### 2.1. Problem definition

In the following, genotype can be a species, an accession, an ecotype or a cultivar. We denote the two genotypes used for grafting as *A* and *B*. Different graft combinations may result in homografts, i.e. stock:scion is *A*:*A* or *B*:*B*, and heterografts, i.e. stock:scion is *A*:*B* or *B*:*A*, see figure 1.

We wish to identify homologous transcripts from the grafted plants that differ in a number of nucleotide positions (SNPs). If we perform RNA-Seq on material from the scion and stock of grafted plants and map the reads onto reference genomes of *A* and *B*, we can expect to find numbers of reads that correspond to *A* and/or *B* for each SNP. See Thieme *et al.* [13] for details on the grafting and RNA-Seq protocols. For each SNP, we denote the total number of reads by *N* and the total number of reads that contain SNPs that correspond to the genotype that is different from the genotype of the sampled tissue by *n* (for short we will say *n* reads map to the other genotype). For instance, if we perform RNA-Seq on tissue taken from a scion of genotype *A*, then those reads that map best to genotype *B* at a certain SNP will be denoted by *n*. If the sample was taken from a homograft *A*:*A* and *n* reads map to genotype *B*, then these *n* must be viewed as ‘errors’ that occurred during the process. Potential error sources include biological variation due to, for instance, mutations or the occurrence of different splice variants, or technical issues during library preparation or polymerase chain reaction (PCR) amplification steps, mapping errors to the reference genome and errors therein, as well as bioinformatic tools and the choice of associated parameters. In electronic supplementary material, figure S1, we describe the relationships between the numbers from RNA-Seq data and the actual reads from each genetic background. The key question is whether the data support the presence of transcripts from the other genotype (e.g. from *B*) in the tissue of the sampled genotype (e.g. *A*) and how many reads we can attribute as being graft-mobile.

We denote the genetic background of the sampled tissue, the local genotype, by ‘1’ (where ‘1’ could be genotype *A* or *B*). We assume that a transcript has a set of SNPs, *S* = {*s*}, for which the RNA-Seq analysis results in data in the form **D** = {*D*_*s*_} = {*N*_*s*_, *n*_*s*_}, where *N*_*s*_ is the total number of reads for SNP *s*, of which *n*_*s*_ map to the other, non-sampled tissue of another genetic background, the distal genotype (denoted by ‘2’).

We define two hypotheses for which we wish to evaluate the statistical evidence:

— *Hypothesis H_1_* states the data can be explained by a statistical model with only one genetic background, i.e. RNA-Seq reads that appear to be from a second genetic background are consistent with sequencing errors, mapping errors, frequencies of somatic mutations, presence of splice variants and any other process that can be expected to introduce mis-assignments in the sampled tissue of genotype 1 → *the data are consistent with the expected biological and technical variance from only one genotype*.
— *Hypothesis H_2_* states that the data are best explained by a statistical model that includes transcripts from distal grafted tissue, i.e. there are RNA-Seq reads from transcripts from a second genetic background that are unlikely to have arisen from the expected variance of genotype 1 → *the data support the presence of RNA-Seq reads from two genotypes and the transcript being graft-mobile*.

**Figure 2.**
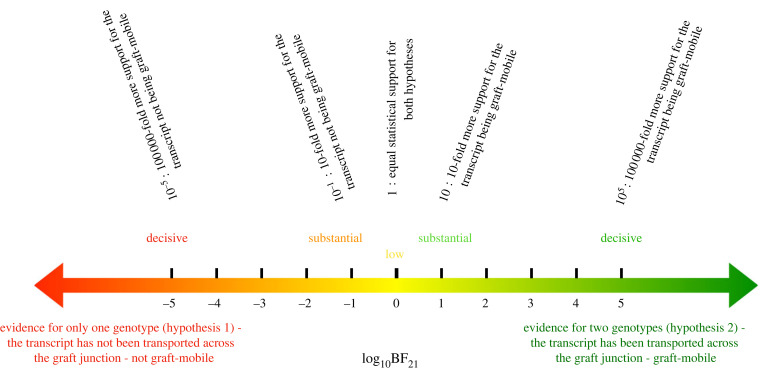
The BF scale and its interpretation. The BF is the ratio of the evidences supporting two different hypotheses. Here, the hypotheses are that the data are consistent with expected biological variation, sequencing and mapping errors (*H*_1_), and the data support the transcript being graft-mobile (*H*_2_). The interpretation of BFs relies on accepted ranges [21,35], as shown in the figure.

Hypothesis 1 thus posits that RNA-Seq reads from only one genetic background are sufficient to explain the data, whereas hypothesis 2 requires that two genetic backgrounds are present. If statistical evidence for two genetic backgrounds is found in the data, then a plausible inference is that transcripts have moved across the graft junction. We can compare hypotheses using the posterior odds ratio, *P*(*H*_2_|**D**)/*P*(*H*_1_|**D**) [21,22]. When both hypotheses, *H*_1_ and *H*_2_, are equally likely *a priori*, *P*(*H*_1_) = *P*(*H*_2_), the posterior odds ratio becomes equal to the marginal likelihood ratio (Bayes factor), BF_21_ = *P*(**D**|*H*_2_)/*P*(**D**|*H*_1_), where *P*(**D**|*H*_1_) and *P*(**D**|*H*_2_) are the marginal likelihoods, also known as evidences. If the ratio of graft-mobile transcripts to non-graft-mobile transcripts were known, then this information could be used as the prior ratio for *P*(*H*_2_)/*P*(*H*_1_). Here, we will assume a 1:1 ratio and use Bayes factors (BFs). Following Jaynes [21], we use the logarithm (of base 10) of the BF. A BF logBF_21_ of 0 means that there is an equal probability for both hypotheses, whereas logBF_21_ > 0 favours hypothesis 2 (graft-mobile) and logBF_21_ < 0 favours hypothesis 1 (errors), see figure 2.

### 2.2. The statistical evidence for a transcript being graft-mobile can be computed from the contributions from its SNPs

The computation of the evidence, *P*(**D**|*H*_1_), requires integration over all parameters associated with *H*_1_, **p**^′^. We assume that these parameters can be specific to each SNP *s* in a transcript, ${\;p}^{\prime }=(p_{1}^{\prime },\;\ldots ,p_{\left|S\right|}^{\prime })$, where |*S*| is the cardinal number of *S*, i.e. the number of SNPs in the transcript. Assuming the parameters are independent between SNPs, the likelihood can be expressed as the product of the likelihoods for each SNP. The evidence for *H*_1_ over all data related to the transcript can thus also be expressed as a product, *P*(**D**|*H*_1_) = ∏_*s*∈*S*_ *P*(*D*_*s*_|*H*_1_), where *P*(*D*_*s*_|*H*_1_) is the evidence for *H*_1_ for data *D*_*s*_ associated with SNP *s*. Analogously we can derive equations for *H*_2_, whereby there may be a different number of parameters associated with each SNP for *H*_2_, ${\;p}^{\prime \prime }=(p_{1}^{\prime \prime },\ldots ,p_{\left|S\right|}^{\prime \prime })$, compared with *H*_1_. The parameters are explained in the following sections and the electronic supplementary material, appendix. Following the aforementioned procedure, we can express the evidence *P*(**D**|*H*_2_) as a product of SNP-based evidence, *P*(**D**|*H*_2_) = ∏_*s*∈*S*_ *P*(*D*_*s*_|*H*_2_). The log BF of hypothesis *H*_2_ (graft-mobile, i.e. there are reads from two genotypes) over *H*_1_ (‘errors’, i.e. reads from only one genotype) can thus be written as the sum over log BFs for each SNP *s* within a transcript (as set of SNPs, *S*),

2.1 $$\log{\mathrm{BF}}_{21}=\sum_{s\in S}\log\frac{P(D_{s}|H_{2})}{P(D_{s}|H_{1})}=\sum_{s\in S}\log{\mathrm{BF}}_{s,21}.$$

This factorization into contributions from each SNP allows us to focus on deriving equations for a single SNP. We then sum these contributions to obtain an overall log BF for a transcript being graft-mobile (*H*_2_) or not (*H*_1_).

### 2.3. SNP-specific error distributions can be inferred from homograft data

As mentioned earlier, we denote the total number of reads over a SNP by *N* and the total number of reads associated with genotype 2 by *n*. If the data came from a homograft or a non-grafted plant, we can assume that *N* − *n* reads were correct and *n* reads have sequencing errors or were mismapped, i.e. there is a SNP-specific error rate that we can infer from the data. A suitable likelihood, Λ(θ), for a two-outcome event is the binomial distribution, where *θ* is the error rate we wish to infer (see electronic supplementary material, appendix).

The conjugate prior to the binomial distribution is the Beta distribution, Beta(*u*_1_, *u*_2_), which results in the posterior having the same functional form,

2.2 $$P(\theta |D)=\mathrm{Beta}(u_{1}+n,u_{2}+N-n)=\mathrm{Beta}(\alpha ,\beta ),$$

where *u*_1_ and *u*_2_ are hyperparameters of the prior and *α* and *β* are the updated Beta distribution parameters of the posterior, see electronic supplementary material, appendix and figure S2.

For a grafting experiment that involves plants with genotypes *A* and *B*, we can infer the following Beta distribution parameters from the homograft data: ($\alpha_{s}^{\mathrm{scion}\;A}$, $\beta_{s}^{\mathrm{scion}\;A}$) and ($\alpha_{s}^{\mathrm{stock}\;A}$, $\beta_{s}^{\mathrm{stock}\;A}$) from *A*:*A*, and ($\alpha_{s}^{\mathrm{scion}\;B}$, $\beta_{s}^{\mathrm{scion}\;B}$) and ($\alpha_{s}^{\mathrm{stock}\;B}$, $\beta_{s}^{\mathrm{stock}\;B}$) from *B*:*B* for each SNP *s*. Examples of inferred posterior distributions for two different priors, error rates and number of reads are shown in electronic supplementary material, figure S3. As expected, the choice of prior loses relevance with increasing read depth.

Replicates can be used to update the posterior distributions. The information content of a dataset remains the same regardless of how it is subdivided or the order in which it is processed, and this is reflected in the mechanics of Bayesian updates [21]. Electronic supplementary material, figure S4 depicts how information is combined (always updating to the latest state of knowledge) within the Bayesian framework described here. As expected, this leads to the same results no matter how the data are split (e.g. into replicates).

### 2.4. The number of reads from transported transcripts can be inferred from heterograft data

**Figure 3.**
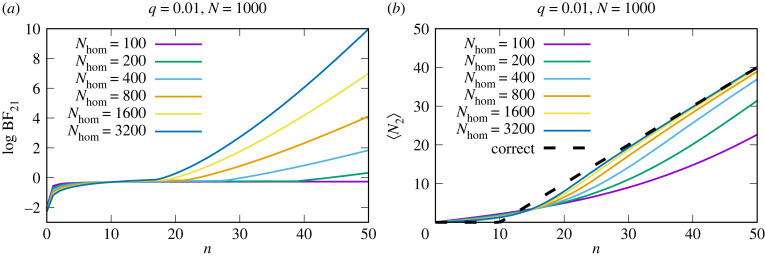
The read depth of the homograft data influences the BFs and the numbers of inferred graft-mobile transcripts. (*a*) It is shown how the BF changes as a function of the number of reads, *n*, that map to the other genotype for different homograft read depths. For an assumed error rate, *q*, of 0.01 and a total number of reads, *N*, of 1000, the expected number of mismatches would be 10. As shown in (*a*), the BF is negative for *n* < *qN* = 10 and moves closer to 0 as *n* approaches the expected number of mismatches. The BF remains negative somewhat beyond the expected number of reads as these low numbers are still consistent with the inferred error distribution. As *n* increases further, the BF becomes positive, favouring the hypothesis that reads may have originated from genotype 2. This change in the BF is shown for different read depths from the homograft data. (*b*) The behaviour of the inferred number of reads from genotype 2, *N*_2_, as a function of *n* and of the read depth from the homograft data, *N*_hom_. With higher read depths from the homograft data and therefore more accurate inferences of the error probability distribution, the estimated number of reads from the genotype 2, 〈*N*_2_〉 approaches the correct value (black dashed line).

As described earlier, we can focus on each SNP individually and then combine the contributions from all SNPs in a transcript, equation (2.1). Given *N* total RNA-Seq reads from sampled tissue of genotype 1 of which *n* contains SNPs associated with genotype 2, we want to know how many reads actually came from genotype 2, *N*_2_, see electronic supplementary material, figure S1. Assuming that the biological and technical variation associated with RNA-Seq analysis will be similar between homografts and heterografts, we can use homograft data as a reference against which to evaluate the heterograft data. As described in the electronic supplementary material, appendix, we can derive the posterior distribution over *N*_2_, *P*(*N*_2_|*D*). From this posterior distribution, the expected ratio of reads from transported transcripts can be computed, $\left\langle N_{2}\rangle =\sum_{N_{2}=0}^{N}N_{2}P(N_{2}|D\right)$ and the ratio as *r*_2_ = 〈*N*_2_〉/*N* for each SNP *s*. For a transcript, the expected ratio is given by averaging over all SNPs. Figure 3 shows how BFs change as a function of *n* and how well *N*_2_ can be estimated for different homograft read depths.

### 2.5. SNP-specific evidence for *H*_1_ and *H*_2_ can be computed from the posterior distribution over *N*_2_

We now show how both *P*(*H*_1_|*D*) and *P*(*H*_2_|*D*) can be computed from the above described distribution *P*(*N*_2_|*D*). Under hypothesis *H*_1_, any reads with SNPs associated with the distal genotype must be considered errors, i.e. there are no transcripts from genotype 2 in the data, *N*_2_ = 0. Under hypothesis *H*_2_, there are reads from transcripts from genotype 2 and *N*_2_ is a natural number. For *N*_2_ > 0, there are *N* possible values for *N*_2_, and we can compare the evidence for each of them against *H*_1_. The uniform prior distribution over *N*_2_ (from 0 to *N*, see electronic supplementary material, appendix) ensures that each instance of *N*_2_ > 0, which corresponds to *H*_2_, will have the same prior probability as *N*_2_ = 0 that corresponds to *H*_1_, thus resulting in an equal prior for *H*_1_ and *H*_2_ and the posterior ratio being equal to the BF. The maximum log posterior odds ratio over all possible *N*_2_ values for a transcript with |*S*| SNPs can now be computed from *P*(*N*_2_|*D*),

2.3 $$\log\frac{P(H_{2}|\;D)}{P(H_{1}|\;D)}=\sum_{s\in S}\log\frac{\max{\left\{P(N_{2}|D_{s})\right\}}_{N_{2}>0}}{P(N_{2}=0|D_{s})}=\sum_{s\in S}\log\frac{P(D_{s}|H_{2})P(H_{2})}{P(D_{s}|H_{1})P(H_{1})},$$

where max is over all positive *N*_2_. Given that this approach has *P*(*H*_1_) = *P*(*H*_2_), the aforementioned equation is equal to the sum of SNP-specific BFs, $\sum_{s\in S}\log{\mathrm{BF}}_{s,21}$, for each transcript. The expression for *P*(*N*_2_|*D*_*s*_) is given in the electronic supplementary material, appendix. The aforementioned equation can be used to assess whether a transcript is graft-mobile from RNA-Seq data, figure 2.

### 2.6. Validation against labelled data

As most predicted graft-mobile mRNAs from RNA-Seq data have not been validated, there is uncertainty regarding their labelling, making an evaluation of the classification performance problematic. We circumvent this problem by creating datasets for which we know exactly which transcripts are assigned to be graft-mobile and which not (see §3). The use of simulated data gives us full control over important parameters such as error rates and read depths while testing the accuracy of our method.

#### 2.6.1. Error rates can be accurately inferred from read counts per SNP in homografts

A first question was how well we can infer the true error rate from simulated data (see §3). Electronic supplementary material, figure S5 shows the inferred error rate, 〈*θ*〉, plotted against the true error rate *q* for a range of *q* from 0 to 1 and for read depth *N* from 10 to 10 000. For low read depths (*N* = 10), the possible number of outcomes is small, leading to a visibly discretized set of inferred error rates with significant variation. For *N* = 100, the inferred error rates match already well to the true error rates. For *N* = 1000 and above, the estimates are accurately defined with high precision. This suggests that with suitably high RNA-Seq read depths (relative to the error rate), we can expect the inferred error rate per SNP to be a fair reflection of the true error rate.

#### 2.6.2. Negative and positive controls are captured well by Bayes factors for individual SNPs

We compare hypotheses by evaluating the evidence for one hypothesis over another. Here, we use logarithm base 10 of the BF for hypothesis 2 over hypothesis 1, log_10_BF_21_, meaning that a value of zero arises when the data give no evidence either way, a negative value when the data is consistent with expected error rates and a positive value if graft-mobile transcripts are present, figure 2.

To investigate whether the BF calculation would correctly assign a negative value for cases where there are only errors (no graft-mobile transcripts), we generated datasets (see §3) for different error rates and different read depths. Data were generated that represent both homograft combinations to infer their posterior distributions for the error rates. Separate data were generated from the same process with the same error rates for each SNP and treated as heterograft data. We found that the BFs from most simulated datasets correctly favour hypothesis 1 that the observed data arose from a process with consistent error rates between homo- and heterograft data. However, as expected, there are several exceptions due to the stochastic nature of the simulations, electronic supplementary material, figure S5. The variability in BFs depends on how well the read depth captures the underlying error rate, for instance an error rate of *q* = 0.01 will not be well represented by a read depth of 100 or less.

After confirming that the BFs perform satisfactorily on negative controls, we next validated the approach on positive controls. We used simulated data for which additional reads from genotype 2 were added (graft-mobile transcripts), thus making them less consistent with the inferred homograft error rates. As anticipated, the closer the number of reads from genotype 2 is to the expected number of errors, the more challenging it is to distinguish graft-mobile transcripts from errors, electronic supplementary material, figure S7. If the error rate for a SNP is *q*, then we would expect to have on average *qN* reads that are errors. The standard deviation of this value is $\sqrt{q(1-q)N}$. If we infer the error rate from the homograft data, we obtain a distribution over the inferred error rate, *θ*, the expectation of which, 〈*θ*〉, is our best estimate for *q*. The detection of reads from graft-mobile transcripts is therefore limited by the available data through the variation (precision) in the inferred error rate. We conclude that the BFs perform well at the individual SNP level, but that false assignments can be expected, in particular for low read depths that fail to represent the underlying error rates.

### 2.7. Combining the evidence across SNPs increases the accuracy of classification

**Figure 4.**
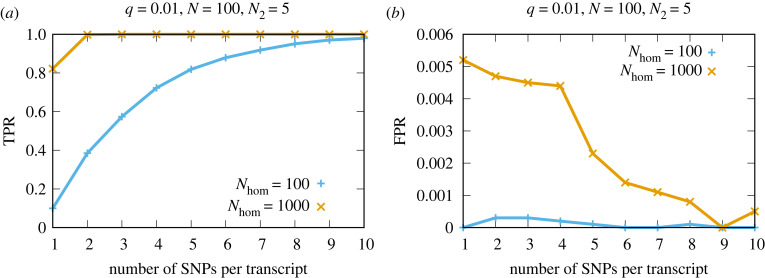
Combining data covering multiple SNPs per transcript enhances the classification accuracy. (*a*,*b*) The result of 10 000 stochastic simulations with a different number of SNPs each with *q* = 0.01, *N*_hom_ = 100, *N* = 100 and *N*_2_ = 5. We assign a transcript as being graft-mobile if log_10_BF_21_ ≥ 1 and say the data are consistent with errors otherwise. TPR is the true positive rate, FPR is the false positive rate (see §3).

A major advantage of the proposed framework is its ability to combine the evidence across multiple SNPs within a transcript, equation (2.1). If the data from several SNPs of a transcript are incompatible with expected errors, then this enhances the evidence of the transcript being graft-mobile. Conversely, if the data from only one out of several SNPs within a transcript are found to deviate from expected errors, and data from the other SNPs are probably errors, then this could result in evidence against the transcript being graft-mobile. We can demonstrate this effect by showing how the distribution of BFs for mobile and non-mobile populations changes as we sum across all of the SNPs in a transcript, electronic supplementary material, figure S8. Interestingly, for anything other than borderline cases of low read depth, this improvement saturates in the simulated data and little is to be gained beyond a SNP number of approximately 3, figure 4.

### 2.8. Comparison with other methods

One key advantage of using BFs is that we are not classifying transcripts *per se* but instead evaluating the evidence for them being graft-mobile, or not. The BFs are thus used to rank our confidence in a transcript being graft-mobile given the data. The strength of the evidence can be assessed using well-established ranges [21,22,35], figure 2.

To compare the presented Bayesian approach with alternative criteria for defining mobile mRNA, we defined and implemented two approaches, Methods A and B, inspired by previous publications [13,18,36]. Method A determines a transcript as graft-mobile based on the number of reads mapping to genotype 2 being above a predefined threshold of 3, in two of three replicates. Method B filters out SNPs with reads mapping to genotype 2 in data from genotype 1 homografts and then assigns SNPs as being graft-mobile when reads in the heterograft data are above 3 in two replicates [36]. So, Method B corresponds to Method A using only pre-filtered SNPs.

**Figure 5.**
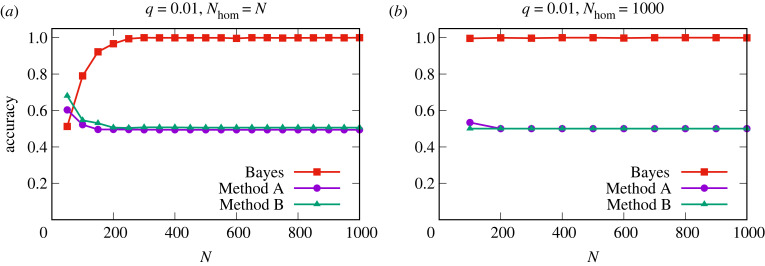
Filtering SNPs based on observed errors or determining mobile transcripts based on absolute read counts leads to poor classification accuracy for simulated data. By using our Bayesian approach (Bayes, red curves), we assign a transcript as being graft-mobile if log_10_ *B*_21_ ≥ 1. Methods A and B are explained in the main text. Here, we have three replicates per transcript. The Bayesian approach sums the evidence over replicates, whereas Methods A (magenta) and B (green) require that two of three replicates show evidence for mobility. Both plots show the accuracy, (TP + TN)/(TP + TN + FP + FN), of each method over 1000 simulated datasets for different read depths, *N*, for an error rate of *q* = 0.01. (*a*) How the accuracy varies for different values of *N* for homograft read depths equal to *N* is shown. (*b*) How the accuracy varies for different values of *N* for fixed homograft read depths of 1000 is shown. The convergence of Methods A and B towards approximately 0.5 is a consequence of the balanced dataset with a 1:1 ratio of transcripts from one genotype (i.e. not graft-mobile) and two genotypes (i.e. graft-mobile), meaning that their performance is essentially little better than random for data similar to the simulated datasets. For an unbalanced dataset, the accuracy could drop below 0.5 for Methods A and B. An analogous analysis for true and false positive rates using data from published RNA-Seq studies is shown in figure 6.

**Figure 6.**
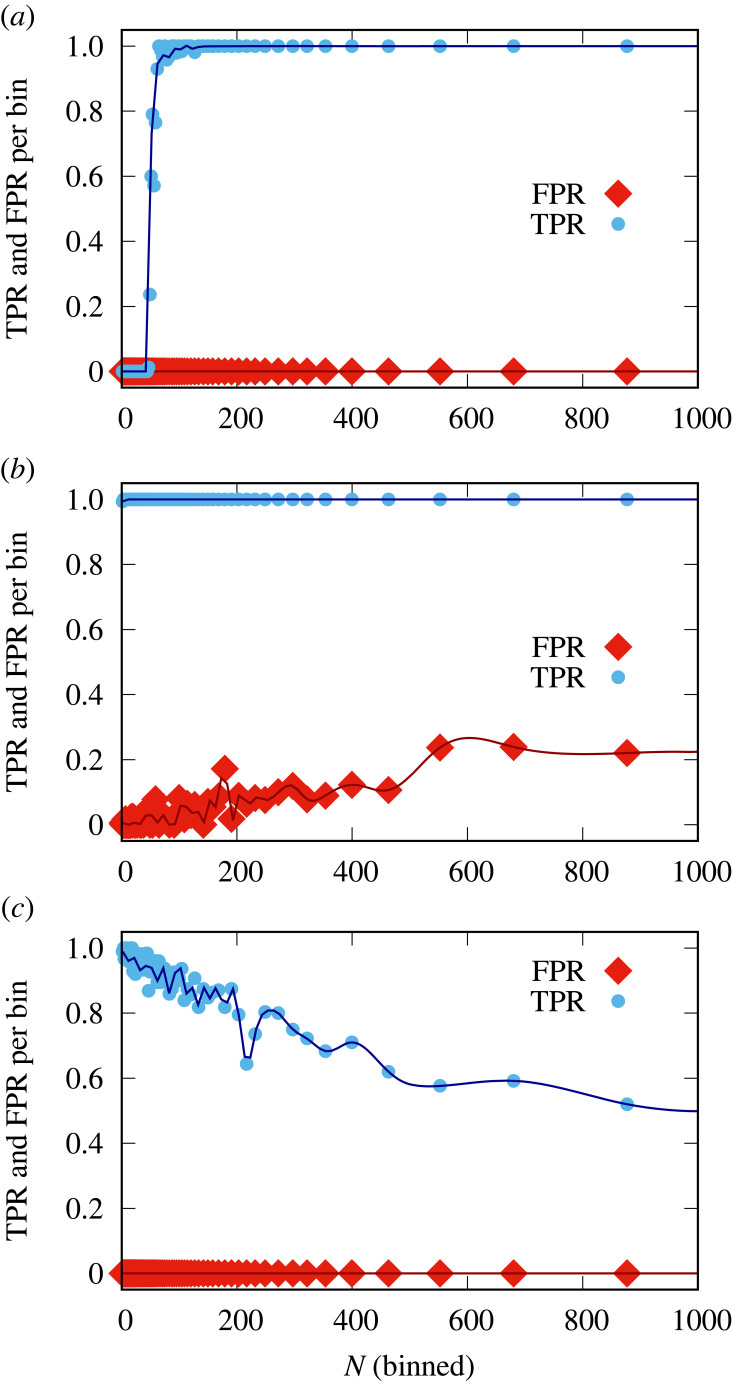
A comparison of methods on artificially generated heterograft data from real RNA-Seq data shows a similar trend in performance to the simulated datasets. The Bayesian approach (*a*), Method A (*b*) and Method B (*c*) are evaluated in terms of their true (TPR) and false positive rates (FPR) for blended real data using a blend factor of *p* = 0.1 (see §3). Only SNPs of comparable read depths between datasets were used and the data were binned for *N* (see §3). TPR and FPR are shown per SNP. Note that for similar read depths at each SNP location between homografts and heterografts, Methods A and B make less mis-classifications than they would for different read depths. Without prior knowledge of the error rates, the Bayesian approach requires sufficient sequencing read depth to build an error model.

**Figure 7.**
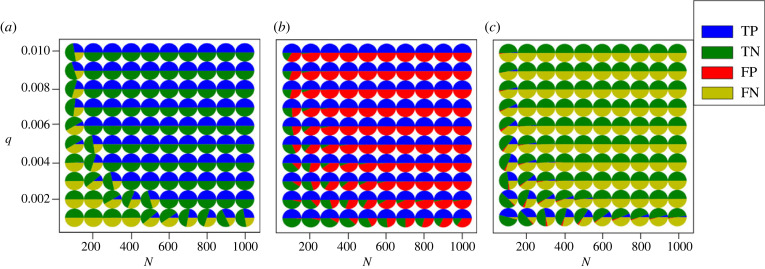
The classification performance of graft-mobile mRNAs depends on the error rates (*q*) and the read depths (*N*) of the RNA-Seq data. The simulation conditions are the same as shown in figure 5. *N* is the same homograft and heterograft data. The numbers of transcripts correctly labelled (TP and TN) based on BFs increases with read depth (*a*). Higher read depths capture the error rates from the homograft data, resulting in clearer separation based on BFs between hypothesis 2 (graft-mobile) and hypothesis 1 (errors). However, as sequencing errors in heterograft data are more likely to arise with higher read depths, we see a rise in false positives (transcripts with errors being incorrectly labelled as being graft-mobile, FP) for Method A (*b*). Conversely, the increase in errors in the homograft data with read depth leads to an increase in false negatives (graft-mobile transcripts being incorrectly labelled as non-graft-mobile, FN) in Method B (*c*). Therefore, both Methods A and B display decreasing classification performance with increasing read depth. Graft-mobile transcripts have been shown to be present in low numbers [18], therefore requiring high sequencing depths to detect them and necessitating methods able to distinguish between errors and graft-mobile transcripts.

Figure 5 shows a comparison of the different methods. We find that as the read depth increases, more and more false positives occur using Method A, which leads to a drop in accuracy. This is because if every nucleotide has an error rate, then higher read depths will result in higher absolute numbers of errors. The conservative nature of Method B means that it has a low rate of false positives, but it is unable to detect graft-mobile transcripts when sequencing errors are present, as they always are, in the homograft data. Method B is thus unable to detect graft-mobile transcripts, unless the error rates are very low, figure 5. Confidently inferring a low error rate per SNP from RNA-Seq data requires a high read depth. Consequently, SNPs with both low read depths and low error rates were not detected in real RNA-Seq data [13], electronic supplementary material, figures S10 and S11. The classification found in the simulated data is also observed using artificial data (see §3) generated from published RNA-Seq data from Thieme *et al.* [13], figure 6. By using a log_10_BF_21_ ≥ 1 (see §3) to classify transcripts as graft-mobile and analysing the same dataset with the Bayesian method delivers both high TPRs and low FPRs, reflected in high accuracy (figures 5–7). The approximately equal read depths between the homograft data and the blended heterograft data reduces the difference in performance between the Bayesian approach and Methods A and B (Methods A and B do not take read depths into account, which leads to a higher mis-classification rate when differences are present). Consistent with the observations made earlier, the Bayesian approach shows an excellent TPR (and accuracy) for simulations with reads from genotype 2 that exceed the expected number of errors in genotype 1, figure 7. The analysis of existing data in terms of sequencing depth and error rate per SNP, electronic supplementary material, figures S10 and S11, shows that experimental data falls into a parameter regime where mis-classifications of Methods A and B can be expected. We conclude that the Bayes factors perform well and significantly better than our implementations of Method A and Method B.

## 3. Methods

### 3.1. Dataset generation

We used two approaches for generating test data, electronic supplementary material, figure S6. The first uses purely simulated data based on the underlying statistical model described later. The second mixes RNA-Seq data from existing homograft experiments to generate an artificial heterograft dataset. Neither approach is likely to capture the full variation and noise inherent in real datasets but have the advantage of having known labels that allow us to evaluate our method in a controlled manner.

#### 3.1.1. Simulation of RNA-Seq data

Simulated datasets are generated based on a binomial distribution with an error rate *q* for each SNP. A random number generator is used to provide stochasticity in line with the expected variance of a binomial distribution. For the homograft datasets, each SNP is assigned a number of reads (*N*) and a value for *q*. A range of values for *N* and *q* were used and are given in the individual figures. For each read, from 1 to *N*, a uniform random number is drawn, and if this number is greater than *q*, then the read is assigned to genotype 1, otherwise to genotype 2, electronic supplementary material, figure S6. The generated reads per SNP thus represent a discrete realization of a stochastic process with a defined error rate, *q*, with *N* reads assigned to genotype 1 and *n* reads assigned to genotype 2. The heterograft data are generated using the same process, but with the addition of further reads, *N*_2_, from genotype 2 that represent mobile transcripts. Different numbers of added mobile transcripts, *N*_2_, are used to evaluate the sensitivity of the method. For measuring the classification accuracy (see below), we use balanced datasets throughout to not distort any of the performance metrics.

#### 3.1.2. Blending real RNA-Seq data

While the simulated data are inherently noisy, they follow an underlying statistical model which may not capture the variability inherent in experimental data. We therefore generated labelled data based on existing RNA-Seq datasets. We took RNA-Seq homograft data from the *Arabidopsis* Col-0 and Ped-0 accessions [13]. We then ‘titrated’ one dataset into the other to create a labelled heterograft dataset, following (1 − *p*) × Col-0 + *p* × Ped-0, where *p* is the blending proportion. For instance, *p* = 0.1 would include 10% of the RNA-Seq reads for each SNP from Ped-0 and add them to the reduced (1 − *p*) reads in Col-0. If a SNP in the Ped-0 dataset had 100 reads assigned to the Ped-0 genotype, then for *p* = 0.1, we would add 10 of these reads to create a graft-mobile transcript. We selected SNPs that had a comparable read depth in Col-0 and Ped-0, electronic supplementary material, figure S6. Note that this approach creates data homograft and heterograft data of similar read depths, providing favourable conditions for Methods A and B. Given the spread and limited data compared with the simulations, we used adaptive binning to provide approximately equal-sized sets to evaluate the performance of the method as a function of read depth *N*.

### 3.2. Bayesian classification criterion

The Bayes factor denotes the evidence provided by the data that supports one hypothesis over another [21,22]. We use these Bayes factors to rank our confidence in a transcript being graft-mobile; however, to evaluate our approach against existing methods that classify into mobile and non-mobile, we need a binary output. Following standard practice [21,35], figure 2, we use a value logBF_21_ ≥ 1 to select hypothesis 2 over hypothesis 1. It is worth noting that we could also determine transcripts for which there is strong evidence for them not being graft-mobile (e.g. those with logBF_21_ ≤ −1). This latter point is important for curating high-confidence datasets for training using positive and negative examples.

### 3.3. True and false positive rates

The classification performance is evaluated using accuracy, and true and false positive rates. Each mRNA is given one of four different labels: an mRNA that is correctly classified as graft-mobile is a true positive (TP); an mRNA incorrectly classified as graft-mobile is a false positive (FP); an mRNA correctly classified as non-graft-mobile is a true negative (TN) and an mRNA that is incorrectly determined to be non-graft-mobile is a false negative (FN). From this, we can compute the true positive rate (TPR) as the proportion of the graft-mobile mRNAs that are classified correctly from all the graft-mobile mRNAs (TP/(TP + FN)), and the false positive rate (FPR) is the proportion of the non-graft-mobile mRNAs that are incorrectly assigned out of all of the non-graft-mobile mRNAs (FP/(FP + TN)). The accuracy is calculated as (TP + TN)/(TP + TN + FP + FN). The accuracy is 0 if no classes are identified correctly (TP = 0, TN = 0) and 1.0 for error-free classification (FP = 0, FN = 0).

## 4. Discussion

Long-distance transport of mRNAs has been shown to be important for plant development and recently also in mammalian systems [37]. A popular method for detecting transported mRNAs in plants involves grafting plants with different genetic backgrounds and using RNA-sequencing techniques to determine whether transcripts have traversed the graft junction. To do so robustly requires consideration of the associated sequencing and alignment errors that are known to occur.

Here, we have presented an approach for distinguishing between expected variation (e.g. biological variation, RNA-Seq errors, processing errors) in RNA-Seq pipelines and putative graft-mobile transcripts. We set up the problem as an hypothesis-testing framework founded in Bayesian inference for which we derived an exact analytical solution. A key, yet unverified, assumption inherent to the current method and other approaches that compare with homograft data is that the biological and technical variance of the RNA-Seq analysis in homografts and heterografts will be similar.

For equal prior probabilities for the hypotheses 1 and 2, the posterior probabilities ratios are equivalent to Bayes factors. Bayes factors are computed for each SNP position based on the associated read counts and then combined into a Bayes factor per transcript. Replicates can be handled analogously. Bayes factors allow transcripts to be ranked based on data supporting their graft-mobility. Higher Bayes factors are indicative of more translocated individual transcripts (the expectation value of which can be computed separately).

We show that RNA-Seq error rates can be accurately estimated using the presented methodology (electronic supplementary material, figure S5) and how the read depth influences the precision (electronic supplementary material, figures S1, S3 and S5) and the inferred number of graft-mobile transcripts (figure 3). Multiple SNPs and replicates can be readily accounted for by summing their contributions (equation (2.1), figure 4 and electronic supplementary material, S4 and S8). We validated our approach extensively with simulations (figure 4, electronic supplementary material, S7–S9). We used simulated RNA-Seq data to allow for accurate quantification of the performance of the method, avoiding the uncertainties inherent in previously published graft-mobile mRNA labels. An additional advantage is that the use of simulated RNA-Seq data allows us to evaluate the performance over a range of parameters. Further validation was carried out using datasets derived from published homograft data [13], figure 6.

The evaluation of different mobile mRNA detection methods showed that approaches using absolute number thresholds of reads per SNP on selected positions lead to high rates of mis-classified transcripts, figures 5–7. Classifying graft-mobile transcripts based on absolute read numbers without consideration of error rates and read depths (Method A) leads to the assignment of mobility to many transcripts for which statistical support is not present (high false positive rate). On the other hand, filtering SNPs with errors in the homograft data (Method B) results in a reduction in the number of detected mobile transcripts (low true positive rate), figures 5–7. The Bayesian method relies on reference data for the estimation of error rates, and the quality and quantity of these data influences its performance. Here, we used a non-informative uniform prior to evaluate the method, but a more suitable choice of prior for the error rates based on existing experimental data and RNA-Seq error analyses [20] would help overcome the limitations arising from low read depths (electronic supplementary material, figure S3).

The approach could be extended in several ways. We have simplified the outcome at each SNP position to map to the two genetic backgrounds used for grafting, resulting in a binomial likelihood. Including errors for all four nucleotides could provide a better description of the overall nucleotide variability, leading to a replacement of the bionomial likelihood by a multinomial distribution. The conjugate prior of the multinomial distribution is the Dirichlet distribution; however, we have not explored how tractable this approach would be to solve analytically in full. A key assumption of the presented approach is that the error rates per SNP are comparable between experiments. This assumption will need to be checked for real data. If necessary the inferred error rate distributions may need to be adjusted to take large deviations into account (e.g. by increasing the spread by reducing the Beta function parameters). Another addition would be to include sequencing quality scores within the framework. These extensions will be the subject of future developments after the careful analysis of existing datasets to assess shortcomings in the presented developments.

To summarize, this contribution presents a Bayesian framework that takes account of read depths, error rates, replicates and multiple SNPs per transcript, providing a powerful means for distinguishing graft-mobile mRNA from RNA-Seq errors. As graft-mobile mRNA are often rare compared with endogeneous mRNA [18], RNA-Seq read depths need to be sufficiently high [36] and chosen with care, and every effort should be made to reduce the risk of contamination [16]. Detecting rare events can be statistically challenging. Error rates from either ungrafted plants or homografts provide useful reference values for the number of sequencing errors to expect for different genomic locations. The higher the read depth of the reference dataset, the better the error rates can be inferred.

## Data Availability

All associated code is freely available from github (https://github.com/mtomtom/baymobil). The data are provided in electronic supplementary material [38].
